# A Survey on Data Compression Techniques for Automotive LiDAR Point Clouds

**DOI:** 10.3390/s24103185

**Published:** 2024-05-17

**Authors:** Ricardo Roriz, Heitor Silva, Francisco Dias, Tiago Gomes

**Affiliations:** Centro ALGORITMI/LASI, Escola de Engenharia, Universidade do Minho, 4800-058 Guimarães, Portugal

**Keywords:** survey, data compression, LiDAR, perception system, autonomous driving

## Abstract

In the evolving landscape of autonomous driving technology, Light Detection and Ranging (LiDAR) sensors have emerged as a pivotal instrument for enhancing environmental perception. They can offer precise, high-resolution, real-time 3D representations around a vehicle, and the ability for long-range measurements under low-light conditions. However, these advantages come at the cost of the large volume of data generated by the sensor, leading to several challenges in transmission, processing, and storage operations, which can be currently mitigated by employing data compression techniques to the point cloud. This article presents a survey of existing methods used to compress point cloud data for automotive LiDAR sensors. It presents a comprehensive taxonomy that categorizes these approaches into four main groups, comparing and discussing them across several important metrics.

## 1. Introduction

Distance measurement systems using the time-of-flight and intensity of light beams have been in existence for almost a century, tracing their roots back to the early 1930s [1]. Initially applied in atmospheric studies [2], the concept of Light Detection and Ranging (LiDAR) was formally introduced by Middleton and Spilhaus in 1953 [3], a little over two decades after its inception. The invention of the laser in 1960 [4] significantly accelerated the development of LiDAR technology. This remote sensing technology employs lasers to calculate distances to objects or surfaces. In the years that followed, LiDAR found widespread applications in various fields, including aerial surveying and mapping, robots, and autonomous vehicles. With the rapid evolution of the automotive industry and the growing desire for self-driving cars, LiDAR technology started to emerge as a key technology for autonomous driving across multiple applications and environments.

Back in 2002, the Defense Advanced Research Projects Agency (DARPA) proposed the Grand Challenge (held in 2004), which consisted of a vehicle competition that aimed to accelerate the development of self-driving technologies and fostering advancements in robotics. Although no robotic vehicle was capable of finishing the course, in its second edition in 2005, five vehicles were able to cross the finish line. The winning vehicle, Stanley [5], took advantage of the LiDAR technology to autonomously navigate the environment. Nowadays, to help define a vehicle’s autonomous capabilities, the Society of Automotive Engineers (SAE) specifies six levels of driving automation. While level zero refers to no driving automation, the following levels progressively increase the car’s autonomous capabilities up to level five, where cars must provide full driving automation with no driver intervention required.

**Figure 1:**
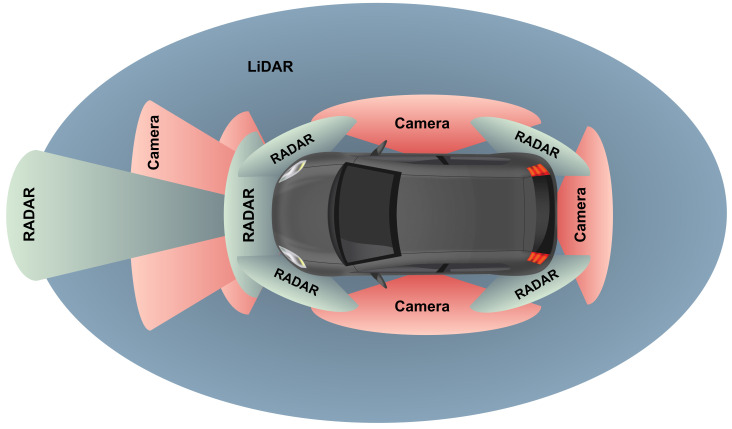
General perception system of an autonomous vehicle.

In order to reach higher SAE levels and accomplish the goal of fully autonomous navigation, a comprehensive perception of the environment is mandatory, which is only possible through the use of multi-sensor configurations [6]. Figure 1 depicts a modern vehicle’s perception system, which includes cameras, Radio Detection and Ranging (RADAR) devices, and LiDAR sensors, among other possible sensor technologies and configurations.

The utilization of these three main technologies presents a diverse set of advantages and drawbacks. For instance, cameras perform very well in object recognition and can provide the best visual information of the surroundings [7]. However, their performance can be reduced in low-light environments and adverse weather conditions, such as fog, rain, and snow. On the other hand, a RADAR demonstrates good reliability in obstacle detection, providing crucial information on their range, angle, and velocity, regardless of some weather conditions [8,9,10,11]. Meanwhile, LiDAR sensors can deliver accurate, high-resolution, real-time 3D representations of the environment around a vehicle [12], allowing for long-range measurements in mostly all-light conditions, making them appealing for autonomous applications. Nonetheless, LiDAR technology still faces some challenges related to specific adverse weather conditions and mutual interference, while struggling to meet the size, weight, power, and cost (SWaP-C) requirements. Regarding its data output, including LiDAR sensors in the perception system may also result in huge volumes of information to be handled during the transmission, processing, and storage operations, which can complicate their interface with resource-limited devices and computer architectures.

One approach to mitigate these challenges is the utilization of data compression techniques to the point cloud. However, the broad landscape of existing solutions includes a wide number of methods and algorithms, which makes difficult the task of choosing one that best suits the final application. To help in understand the existing compression methods available in the literature, this article contributes with:

1. A literature review on state-of-the-art compression methods applied to LiDAR point cloud data and suited for automotive applications;
2. A comprehensive taxonomy with four main categories and several sub-groups, followed by a discussion of the technical aspects of each group and respective methods;
3. A qualitative comparison between the different methods regarding their main features and other important metrics including real-time and compression performance.

The remainder of this article is organized as follows: Section 2 presents the concepts, applications, and challenges behind automotive LiDAR sensors; Section 3 introduces the challenges associated with compressing LiDAR data, the metrics used to evaluate the methods, and the proposed taxonomy; Section 4, Section 5, Section 6 and Section 7 discusses each proposed category and their respective compression methods; Section 8 discusses and provides a qualitative comparison between all contributions, and includes a future perspective on which methods will better adapt to keep up with the ongoing evolution of LiDAR technology; and finally, Section 9 concludes this article.

## 2. Automotive LiDAR

**Figure 2:**
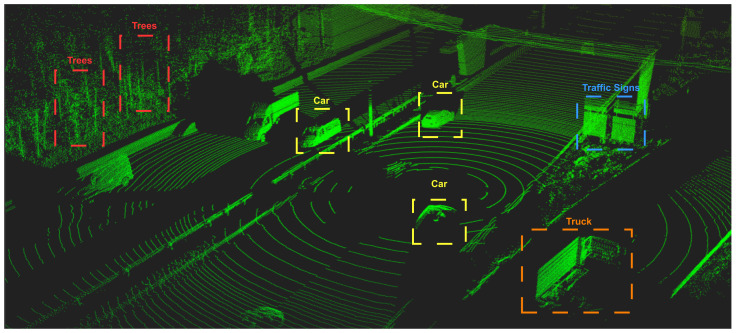
LiDAR point cloud data [13].

The integration of more sensors in the perception system of the car has become pivotal in the automotive industry as more applications require precise information about the surroundings of the vehicle. LiDAR sensors, although relatively new, bring a plethora of advantages when compared with more mature technologies. It is composed of a transmitting unit that uses laser diodes to send light pulses across the Field of View (FoV), measuring the round-trip-time of the light pulse traveling to the target and back within the LiDAR’s range. By its turn, the receiver unit is responsible for collecting the reflected light and processing the received data, which may include the distance traveled by the light pulse and the light intensity. Finally, the sensor utilizes the collected information to create a 3D map of the environment, designated as point cloud, with all the detected points surrounding the sensor. Figure 2 illustrates a LiDAR point cloud captured by a sensor on the top of a car in a highway environment, where distinct objects and shapes, such as cars, trees, road boundaries, and traffic signs, can be perceived by the onboard applications.

### 2.1. LiDAR Applications

By creating the high-resolution 3D visualizations of the surrounding environment in real-time and with great precision, LiDAR sensors become a key instrument in several applications, including critical and safety-related tasks such as collision avoidance, drivable area detection, pedestrian detection and recognition, traffic signs monitoring, and Simultaneous Localization and Mapping (SLAM).

**Collision detection and avoidance:** It represents a critical requirement for autonomous systems, as it involves the identification and detection of both static and moving objects surrounding the vehicle. By accurately detecting the speed, direction, and position of these objects, the safety of the vehicle and its passengers can be effectively preserved [14,15,16].
**Drivable area detection:** LiDAR sensors can assist in detecting the road and the drivable area, where high-level algorithms are able to accurately identify road boundaries, markings, lanes, and curbs, aiding in a correct evaluation of the road and ensuring efficient navigation of the vehicle [17,18,19]. To better perform these tasks, a ground segmentation step can be applied to the point cloud data [20], which enhances the subsequent identification of environmental features.
**Road users detection and tracking:** Prioritizing the safety of both the passengers inside the vehicle and individuals outside is paramount, and LiDAR sensors can assist high-level applications in the detection and tracking of road users, such as pedestrians, and bicycle and motorbike riders [21,22,23]. Such features enable autonomous vehicles to make informed decisions and take appropriate actions to avoid collisions.
**Road signs monitoring:** The use of LiDAR sensors in monitoring road signs related to traffic regulations brings significant advantages [24,25,26], mainly due to the ability to detect their highly reflective surfaces. This feature can significantly enhance road safety and facilitate efficient traffic management, underscoring the essential role that LiDAR sensors play in ensuring the reliability and intelligence of autonomous systems.
**SLAM:** This technique is an extensively researched field in robotics that consists of constructing real-time localization maps using perception data. Its application has extended to autonomous vehicles, leveraging the vast amount of 3D information generated by LiDAR sensors [27]. Typically, traditional odometry techniques combine data from several sensors to estimate the vehicle’s position relative to an initial reference point. To accommodate the high data rates of LiDAR sensors, approaches like LOAM [28] prioritize frequent odometry processing while reducing mapping frequency to ensure real-time performance.

### 2.2. Challenges

Despite the significant advantages of incorporating LiDAR sensors into modern perception systems, several technological challenges still remain, motivating researchers and manufacturers to continue studying and investing on improving this technology. Contributing to this field of study is not straightforward, as it can be hard to recreate real-world situations to deploy and test LiDAR sensors. Nonetheless, some research centers already developed expensive facilities supporting complex road and environmental setups to validate and benchmark different driving environments. For instance, LIBRE, considered the first benchmarking and reference LiDAR dataset, tested ten sensors in three different environments and configurations such as static targets, adverse weather, and dynamic traffic [29,30]. Gomes et al. also proposed a testing platform to rapidly test and validate LiDAR sensors by analyzing only their point cloud output [31]. The testing system is able to benchmark a LiDAR sensor in a controlled environment to recreate the expected driving conditions to which such devices are normally subjected. Taking advantage of these solutions, some research areas focus on making LiDAR more accessible and efficient by exploring the development of cost-effective, reliable [32], and reduced size sensors for better vehicle integration [33], while others address issues related to optimizing LiDAR performance across different environments and applications. These improvements include enabling LiDAR operation under adverse weather conditions, such as rain, snow, and fog [34,35,36], as well as enhancing data transmission, processing, and storage capabilities [37].

## 3. Automotive LiDAR Data Compression

For a better understanding of the environment, it is required that sensors provide high-resolution point clouds, in real-time. However, this increases the hardware requirements for the sensor itself, as well as its demands for high-speed interfaces and processing architectures for transmitting, handling, and storing LiDAR point cloud data, which can be particularly challenging in embedded perception systems with limited memory and bandwidth resources. Some approaches to mitigate these challenges include the optimization of the interface used by sensors (minimizing the latency and increasing the throughput), and the deployment of data compression algorithms. Cunha et al. [38] propose an Ethernet interface solution for data packet decoding and reconstruction compatible with different LiDAR sensors. By decoding the data packets and using hardware-assisted algorithms to translate points between different coordinate systems, data transmission can be improved without losing point cloud information. Nonetheless, some top-class sensors, such as the Velodyne VLS-128, can produce up to 9.6 million points per second, which further demands for compression algorithms to optimize the streaming and storage applications.

Storing the data generated by LiDAR sensors within an automotive environment provides many advantages, such as environmental classification, SLAM applications, and the creation of datasets, extremely useful for machine learning algorithms and for point cloud data processing without requiring the sensor being installed in the real-world environment. Depending on the sensor and the setup used, storing LiDAR raw data is not always feasible. Therefore, the utilization of data compression can be useful to identify and eliminate data redundancies of spatial and temporal nature [37], resulting in less data to be transmitted, processed, and stored.

### 3.1. Proposed Taxonomy

**Figure 3:**
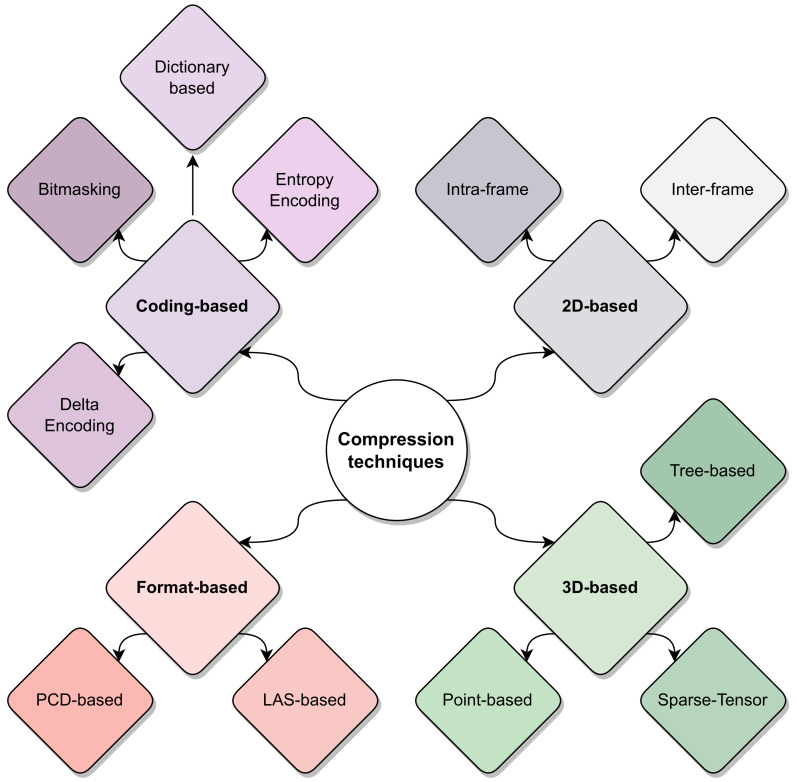
Proposed taxonomy for LiDAR data compression methods.

Point cloud data compression has become a prominent topic in several research topics, ranging from Virtual Reality (VR) applications featuring dense point clouds, to automotive scenarios where the point clouds (generated by a LiDAR sensor) are usually sparse and cover a wider 3D area. Within the scope of this article, this survey only addresses contributions that specially target automotive LiDAR sensors, whether through real implementation, dataset analysis, or sensor/environment simulation. Figure 3 summarizes the proposed taxonomy for classifying state-of-the-art compression techniques, organizing them into four main groups: (1) **Coding-based compression**, which includes fundamental low-level compression algorithms applied to raw data; (2) **Format-based compression**, which uses well-known and standard LiDAR compression formats; (3) **2D compression**, which include methods that employ a 2D projection of the point cloud to apply image/video codecs; and (4) **3D compression**, which summarizes the approaches using 3D space partitioning to decompose the LiDAR data before being compressed.

### 3.2. Performance Metrics

Among the various metrics used to assess a compression technique’s performance, the most common are the Compression Ratio (CR) and the processing time required to complete the compression process. The CR compares the size of the point cloud data before and after the compression being applied and it can be obtained with Equation (1), where the Uncompressed_Size corresponds to the original point cloud size, and the Compressed_Size is the size of the point cloud data after being compressed. Therefore, a higher CR value indicates a bigger size reduction.

(1) $$CR=\frac{Uncompressed\_Size}{Compressed\_Size}$$

Similarly to the compression ratio, the bits per point (bpp) can also be used to evaluate the compression performance by quantifying the amount of data after the compression. The bpp, as shown in Equation (2), is the total number of bits after compression (Total_Compressed_Bits) divided by the number of input points (Total_Input_Points). Contrary to the compression ratio, a lower value of bpp indicates better compression performance.

(2) $$bpp=\frac{Total\_Compressed\_Bits}{Total\_Input\_Points}$$

Point-to-plane Peak Signal to Noise Ratio (PSNR) can be also used to measure the quality of decompressed point clouds, especially when compression methods discard information to achieve higher compression ratios. The PSNR is usually expressed as a logarithmic value using the decibel scale. As Nardo et al. [39] describe, let *P* be the original point cloud, |P| be the total number of points contained in the original point cloud, p∈P be one point in the original point cloud *P*, $\hat{P}$ be the reconstructed point cloud, $q\in \hat{P}$ be *p*’s nearest neighbor in the reconstructed point cloud, $\hat{P}$, $n_{q}$ be the surface tangent in $q\in \hat{P}$, $\langle p-q,n_{q}\rangle$ be the projection of p−q vector on $q\in \hat{P}$, and $\theta_{P}^{*}$ be the peak value in the original point cloud, the PSNR can be calculated with Equation (3), being the Mean Squared Error (MSE) obtained with Equation (4). It can be equally important to evaluate a technique’s time performance or latency since compressing LiDAR data in real-time may be a critical requirement in autonomous driving applications that require decision-making purposes. For example, a technique used to compress data and store data from a LiDAR sensor operating at 10 Hz must be capable of completing the full operation within 100 ms to meet real-time processing requirements and to avoid discarding any frames.

(3) $$PSNR_{P\to \hat{P}}=10\log_{10}\left(\frac{{\left(\theta_{P}^{*}\right)}^{2}}{MSE_{P\to \hat{P}}}\right)$$

(4) $$MSE_{P\to \hat{P}}=\frac{1}{\left|P\right|}\sum_{\forall p\in P}{\left(\langle p-q,n_{q}\rangle \right)}^{2}$$

Aside from these metrics, compression techniques can be classified based on the type of compression applied and their application goal. Firstly, the compression type can be divided into lossless and lossy techniques. A lossless approach involves identifying and eliminating statistical redundancy while completely preserving the original information. Contrarily, a lossy method reduces the data size by removing information through a quantization process [40], making impossible the task of reconstructing the original data [41]. The application goal usually determines whether the compression technique is adequate for data streaming, storage, or both. Streaming compression techniques are mainly used by real-time applications where minimizing data transmission between devices is crucial. In contrast, storage-based approaches are often required to compress LiDAR data for future processing and offline visualization. Since most storage applications usually require large amounts of data to be recorded, they use algorithms with better compression ratios in order to save memory resources. However, such algorithms can be more computationally hungry than others used by streaming compression applications.

## 4. Coding-Based Compression Algorithms

There are several coding-based algorithms that can be used to compress LiDAR point clouds, either targeting raw data sent by the sensor or further used by higher-level compression methods. Generically, they handle data as a stream of bits, grouping and reorganizing them into smaller chunks of compressed information. The most common methods are based on entropy coding, dictionary-based coding, delta encoding, and bitmasking, as summarized in Table 1.

### 4.1. Entropy Encoding

Entropy encoding consists of a lossless data compression approach that exploit statistical redundancies to reduce the data size [48]. Among the most popular, Huffman coding [49], arithmetic coding [50], and Golomb coding [51] have been widely used on image and video applications. The Huffman coding scheme involves assigning each data symbol a variable-length code based on its probability of occurrence, with more frequent symbols receiving shorter codes and less frequent symbols receiving more extended codes. This approach results in a more efficient data representation, reducing the overall file size. On the other hand, arithmetic coding encodes the entire input stream into a single value by iteratively refining intervals assigned to each symbol according to their probabilities. Despite its greater complexity and execution time, arithmetic coding represents an improvement over Huffman coding.

Golomb coding is a lossless data compression method designed for encoding distributions in which small values are more frequent in the input stream. It is described in Equation (5), where $G_{m}\left(input\right)$ is the Golomb code for the input data using the parameter *m*, *q* is the quotient of the input divided by *m*, *r* is the remainder of the input divided by *m*, $binary_{l}ength$ represents the number of bits required to represent *m*, and binary(r) represents the binary representation of the remainder *r*. The parameter *m* is used to control the trade-off between the length of the codewords and the frequency of occurrence of different values. A larger *m* value results in longer codewords for smaller values of the input, and shorter codewords for larger values of the input.

(5) $$G_{m}\left(input\right)=q\times binary\_length\left(m\right)+binary\left(r\right)$$

The Golomb–Rice coding [42], which is derived from the original Golomb coding scheme, restricts the configurable parameter *m* to the power of two values, making it more efficient in reducing the computational requirements. To the best of our knowledge, Golomb–Rice coding is the only entropy encoding method that has been directly evaluated on LiDAR raw data, while Huffman and arithmetic coding are typically used in intermediate stages of high-level algorithms.

### 4.2. Dictionary-Based and Delta Encoding

**Figure 4:**
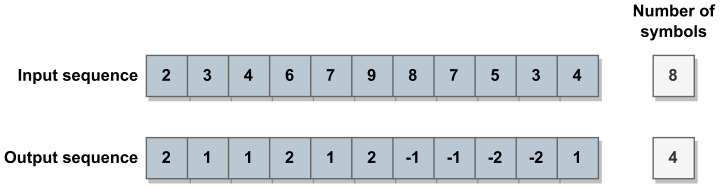
Delta encoding.

Dictionary-based methods identify and store recurring structures within the input data, making them especially efficient when data includes substantial pattern repetition. When an input data sequence aligns with a pattern already stored in the dictionary, only the offset, the length, and a pointer to the matched pattern are coded in the final bitstream, reducing the final data size. Thus, the number of matches and the match length directly influence the overall compression ratio. Nonetheless, each dataset has a unique dictionary, which must be stored alongside the compressed data to allow the decoding step. The work by Maksymova et al. [45] suggests the utilization of the Lempel–Ziv–Markov chain algorithm (LZMA) with LiDAR data. The LZMA is a lossless dictionary-based compression approach that uses the LZ77 algorithm [52] and can be used in different applications that require both the streaming and storage of LiDAR data. This algorithm comprises three stages: (1) delta encoding; (2) sliding dictionary encoding; and (3) range encoding. In the delta encoding stage, a symbol is replaced by the difference between its value and a reference value. As depicted in Figure 4, this state tends to increase the efficiency of the resulting sliding dictionary since it reduces the number of distinct symbols in a sequence. Next, the sliding dictionary applies the LZ77 algorithm, utilizing a search buffer with a defined range to identify patterns in preceding data. It outputs the offset to the matched data, the length of the match, and the next input symbol. The final stage involves the range encoder [53], an entropy encoding method based on the arithmetic encoder [50].

In a standard delta encoding phase, the delta values can range from a negative number up to its positive value minus one. However, if the calculated difference is bigger than the maximum positive value, it generates an overshoot, and the current value is stored with a new codeword. Makysmova et al. [43] propose an extended delta encoding method. They expand the standard delta encoding algorithm to handle the overshoot detection, making it reach compression ratios at least twice as high as the standard approach. The evaluation of this method also included a different version of a delta encoding proposed by Liang et al., where the standard method was improved with symmetric and segmented properties to improve energy efficiency in wireless sensors [46].

### 4.3. Bitmasking

Caillet and Dupuis propose a different compression approach for efficient Vehicle-to-Vehicle (V2V) or Vehicle-to-Infrastructure (V2I) applications that explore a quantization process [47]. Their proposed method involves applying a bitmask to the raw binary data from a LiDAR sensor, which reduces the number of bits by zeroing the least significant bits. This process enhances the efficacy of the next compression techniques, such as LZMA, GZIP, or BZIP2, which take advantage of the zero patterns generated by the bitmask. Moreover, the quantity of nullified bits can be adjusted to achieve higher compression ratios as long as the point cloud retains an acceptable accuracy.

## 5. Format-Based Compression Algorithms

With the LiDAR’s mass adoption in fields such as airborne surveying and mapping for topography, entities like American Society for Photogrammetry and Remote Sensing (ASPRS) started to develop standards to facilitate the interchange of LiDAR data. One of the first standards widely used and targeting airborne LiDAR is LAS [54], which, due to the large datasets generated by this kind of application, started to include lossless compression algorithms to reduce the data size, resulting in the LASzip [55] and LAScompression [56]. They both use prediction coding to compress blocks of LiDAR points, predicting the attributes of a new point from a past or groups of past points. The predicted deltas are then compressed using entropy encoding. Despite their popularity, these algorithms were especially designed to handle airborne LiDAR data, which is collected by sensors with different characteristics than automotive LiDAR sensors. Thus, their application in automotive applications becomes limited.

Another popular representation for point clouds is the PCD format, the data representation used by the Point Cloud Library (PCL) [57]. The PCL is an open source library that provides algorithms and data structures for handling the 3D point cloud data collected by a LiDAR sensor. It supports a wide range of point cloud functionalities, including filtering, segmentation, object recognition, 3D-to-2D projection, and others. More importantly, it provides some techniques that can be used for compressing point clouds, like octree-based compression, range-image conversion and compression, and the PCD file format native to PCL. The PCD can store point cloud data in three different types: ASCII, binary, and binary compressed. Beek et al. [58] evaluated the LASzip and the PCD file with a Velodyne HDL-32, a sensor widely used in automotive applications. Their evaluation, with results presented in Table 2, shows that these formats could not achieve good bpp ratios.

## 6. Two-Dimensional-Based Compression Algorithms

**Figure 5:**
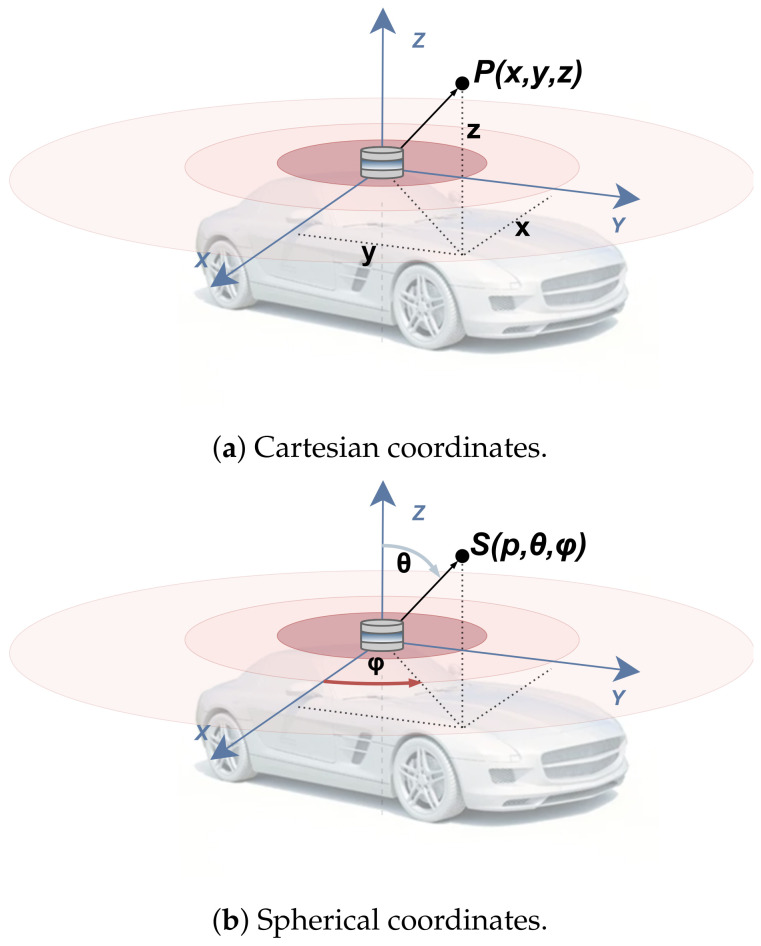
Three-dimensional coordinate representations.

Compression algorithms based on 2D representations focus on compressing images that represent point clouds. First, the 3D point cloud is converted into a 2D structure, which stores either points represented by the Cartesian coordinates system (x,y,z) or the spherical coordinates system (ρ,θ,φ), as illustrated in Figure 5.

**Figure 6:**
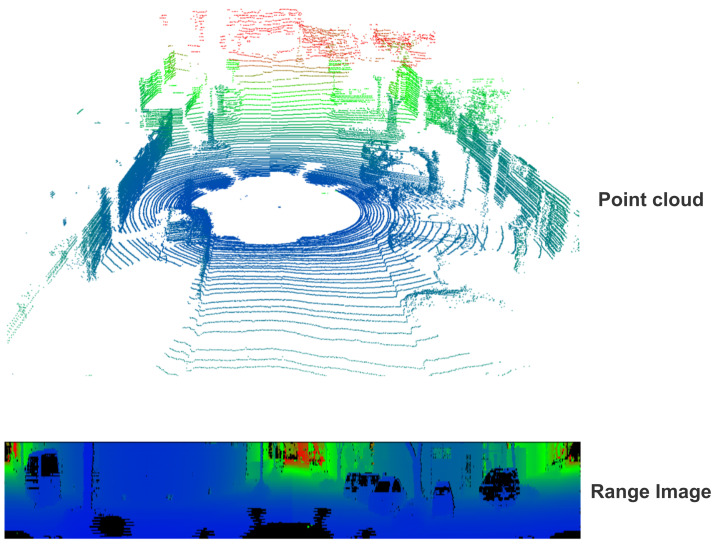
Point cloud to range image conversion.

For compression using Cartesian projections, the 3D coordinates (x,y,z) are assigned either to three single-channel grayscale images (each image representing one Cartesian axis) or combined into a single three-channel RGB image, where each color channel represents one coordinate. In the case of spherical coordinates’ representation, the 2D projection can follow two different approaches. The first option involves projecting the spherical coordinates in the same way as the Cartesian system but with (ρ,θ,φ) values (representing a lossless way to store and recover 3D data). In contrast, the second method involves a lossy projection, where the spherical 3D coordinates are used to calculate the point’s *x* and *y* values within a 2D image. With this method, each point’s range (ρ) is then stored as the image pixel value, creating a representation known as Range Image (RI), as depicted in Figure 6. Once the point cloud data are transformed into a 2D image, efficient encoding techniques, either using standard image compression algorithms or image codecs, are used to compress data. These techniques are generally classified into two different categories: intra-frame (summarized in Table 3) and inter-frame compression (summarized in Table 4). The former, also referred to as spatial compression, aims to minimize the redundancy present in a single image frame, while the latter, also known as temporal compression, is designed to encode the variations across successive frames.

### 6.1. Intra-Frame Compression

Among the several industry standards available for image compression, two of the most widely used methods for compressing image data are the Joint Photographic Experts Group (JPEG) and the Portable Network Graphics (PNG) formats [59]. The JPEG format is a widespread method of lossy compression for digital images, mainly used to store photos. The compression process involves converting the image from RGB to the YCbCr color space, segmenting it into blocks, and then applying a discrete cosine transform (DCT) to transition from spatial to frequency domain data. This transformation highlights significant features while allowing for the reduction in less critical information. Next, the quantization reduces the precision of the DCT coefficients, which is the primary source of compression. The final step encompasses a lossless data encoding algorithm such as the Huffman coding [49]. Since the original JPEG algorithm is inherently lossy, some lossless/near-lossless variants were also developed, such as the JPEG 2000 [60] and the JPEG-LS [61].

**Figure 7:**
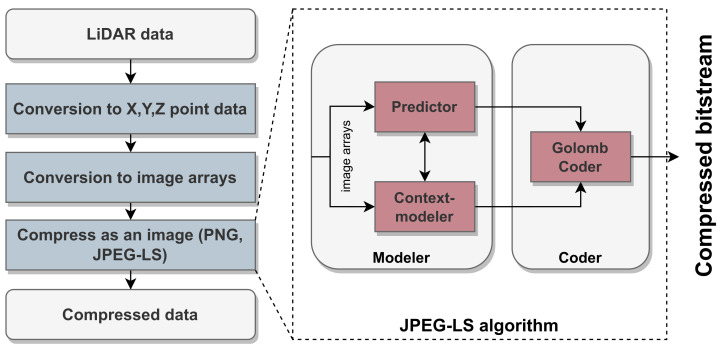
Image-based 3D point cloud compression.

The JPEG 2000 [60] improves the original standard using a discrete wavelet transform (DWT) step instead of a DCT, which increases the overall compression ratios. It also provides lossy and lossless compression flexibility according to the application’s needs. Regarding the JPEG-LS [61], which is derived from the Low Complexity Lossless Compression for Images (LOCO-I) algorithm [62], it provides a simple, yet efficient, implementation. JPEG-LS consists of two basic steps: the first step involves a context-modeler and a predictor that estimates the pixel values based on their surroundings. The following step applies a Golomb–Rice encoder to compress these predictions efficiently. Figure 7 depicts the encoding steps of image-based algorithms to compress point cloud data.

The PNG is a widely used image encoding algorithm that is able to provide lossless compression, preserving the original image information that can be fully recovered in the decompression step. The PNG algorithm begins with a prediction stage that estimates pixel values based on neighboring pixels. This is followed by the DEFLATE algorithm [63], a combination of the LZ77 and the Huffman encoding schemes. Several research studies [58,64,65] have explored the utilization of PNG, JPEG, and their variations for compressing LiDAR data, comparing their results with other image-based and non-image-based compression algorithms to showcase the applicability of these approaches with LiDAR sensors. Aiming at enhancing the compression ratios, Youguang et al. [66] studied the Cartesian-to-cylindrical projection as an alternative to the Cartesian and spherical coordinate systems to represent the LiDAR point cloud. Their proposal includes a regularized representation considering the LiDAR’s mechanical structure and acquisition pattern, specially tailored to LiDAR data compression.

Other methods use segmentation algorithms to create regions of interest within the point cloud before performing data compression. This way, different approaches can be used in specific parts of the point cloud, applying near-lossless compression in the most important information while using high compression and lossy algorithms to the remaining data. X. Sun et al. [67] proposed an approach based on configurable clustering for lossy/lossless compression depending on the selected algorithm. Since a LiDAR sensor can generate many outlier points during the scanning of the environment, this approach applies an outlier removal filter before creating a range image from the LiDAR data. Then, it uses the 3D-HEVC [68] range image-based clustering segmentation method to separate the data into ground and objects. The object areas are later classified based on their geometry using a prediction method. Finally, the compression step is applied either using a lossless technique, such as BZip2 and LZ4, or a lossy technique, like JPEG and JPEG 2000. The work proposed by Chen et al. [69] creates two RIs by segmenting the point cloud into ground points and non-ground points, applying different degrees of sparse sampling to remove redundancies. Finally, the two RI are then compressed using JPEG-LS.

Alongside traditional image compression methods, machine learning techniques also started to emerge in the literature due to their ability to extract features and patterns from data. Tu et al. [70] proposed an image-like Recurrent Neural Network (RNN)-based approach to compress RIs created from LiDAR sensors, also including a residual block structure in the decoder to improve decompression performance. Similarly, Wang et al. [71] applied an entropy model directly to RIs. Other approaches use learning-based semantic segmentation steps in their algorithms. For instance, Ref. [72] uses the RangeNet++ algorithm to retrieve important segments in the RI before compressing them with traditional compression algorithms [73,74] or with learning-based ones [75]. With the goal of improving LiDAR 2D representations, Luo et al. [76] propose a learning-based model-agnostic framework to convert raw data into spherical coordinates. This framework can be used as a pre-processing step for other methods, enhancing their performance.

**Table 3:** Summary of 2D-based intra-frame compression methods.

Category	Method	Type	Main Features	Setup	Dataset	Performance	Source Code
Traditional	PNG (1997) [59]	Lossless	Applies PNG to range images from LiDAR point clouds	Not disclosed	Velodyne and Ibeo sensors	bpp: 7.5–15.2 [58] PSNR: 111 [39]	Open source
	JPEG-LS (2000) [61]	Lossless	Applies JPEG-LS to range images from LiDAR point clouds	Not disclosed	Velodyne and Ibeo sensors	bpp: 6.4–22.4 [58] PSNR: 110 [39]	Open source
	CLUSTER (2019) [67]	Lossless/Lossy	Uses the shape of RI’s segmented regions to feed the prediction module	Intel Core i5-6300HQ with 4GB RAM	KITTI	CR: 4.83–30.21	Not disclosed
	SC-CSS (2021) [69]	Lossy	Compresses segments of non-/ground points using a combination of RI and 3D representations	Not disclosed	Velodyne HDL-32E sensor	bpp: 6	Not disclosed
	RAI IC (2022) [65]	Lossless	Uses standard image compression methods on images created from range, azimuth, and intensity	Not disclosed	Velodyne VLP-32C sensor	bpp: 10–17	Open source
	Cylindrical Pred. (2023) [66]	Lossless/Lossy	Deploys a prediction scheme on a Cartesian-to-cylindrical projection for spinning LiDARs	Not disclosed	KITTI	-	Not disclosed
Learning	2D RNN with RB (2019) [70]	Lossy	Uses a RNN with Residual Blocks on range image-based matrices	Intel Core i7-7820X w/ Nvidia GeForce GTX 1080	Velodyne HDL-32 sensor	bpp: 2.04–4.046	Not disclosed
	HSC (2021) [73]	Lossy	Applies Draco [77] on semantic segments provided by RangeNet++ [72]	Intel Core i7-7700K w/ Nvidia TITAN RTX and 32GB RAM	SemanticKITTI	bpp: 0.2–14 PSNR: 30–70	Not disclosed
	RIC-Net (2022) [71]	Lossless/Lossy	Applies a three stages end-to-end range image-based entropy network	Intel Core i7 w/ Nvidia GeForce GTX 1080Ti	KITTI, Oxford and Campus16	bpp: 4.1	Not disclosed
	R-PCC (2022) [75]	Lossless/Lossy	Applies real-time sampling segmentation and point-plane mixing modeling to RI	Not disclosed	KITTI, Oxford and HKUSTCampus	bpp: 1.15–5.67	Open source
	SPR (2022) [74]	Lossy	Encodes labels, predictions, and residuals from RangeNet++ [72] RI segments	Intel Core i7-7700K w/ Nvidia GTX 1080Ti	SemanticKITTI	bpp: 6.3–7	Not disclosed
	SCP (2023) [76]	Lossless/Lossy	Offers a framework to convert raw data to spherical coordinates	2 AMD EPYC 7742 and 8 Nvidia A100	Ford and SemanticKITTI	-	Not disclosed

### 6.2. Inter-Frame Compression

Similarly to a video sequence, LiDAR point cloud data consistently exhibit a strong correlation between frames (considering a full environment scan), especially when vehicles are moving at low speeds. Therefore, some approaches explore video-based codecs applied to the RI resulting from the point cloud. For instance, Nenci et al. [78] suggest a compression method to allow range-data streams provided by a Velodyne 3D LiDAR sensor to be sent over a low bandwidth network. The method uses the H.264 video codec, a video standard that uses a motion-compensated inter-frame prediction, intra-frame prediction, transformation and quantization, and entropy coding. Similarly, Heo et al. [79] further explore this concept by applying the H.264 video codec to lossy RIs with the primary goal of achieving low latency results. Using a different approach, Nardo et al. [39] applied the LZW [80] algorithm and MJ2 [60] to a stream of RIs. While the LZW is a simple dictionary-based algorithm focused on compression speed, the MJ2 consists of a video codec that encapsulates motion sequences using JPEG 2000 images. Similarly, Tu et al. [81] developed a compression method that combines a RI and video codecs. However, instead of applying the compression method directly to the RI generated from each point cloud frame, different types of RIs are used. The first type, *Log range images*, involves converting the distance information into a logarithmic value, similarly to popular strategies used in audio encoding. The second type, *layered range images*, consists of combining sequences of standard RIs into a range image matrix and then re-cutting the matrix by its rows to generate layers.

Taking advantage of the 3D characteristics present in the LiDAR point cloud data, Tu et al. [82] proposed a lossy video compression algorithm that uses location and orientation information in the compression step. Each point in the LiDAR packet is represented by an ID, rotation position, and distance. All three components are compressed separately using several MPEG and DPCM strategies. In a more recent work proposed by Tu et al. [83], the method includes an improved searching strategy, motion analysis, and better encoding schemes. Sun et al. [84] also improved their work [67] with an inter-frame strategy based on registration and lossless compression on residuals. Feng et al. [85] suggest a lossy technique for compressing point clouds involving the encoding of planes formed by the points in the point cloud. Taking advantage of the significant overlap between consecutive point cloud frames in physical space, it allows for spatially encoded planes to be re-used for encoding the temporal stream.

Deep-learning-based approaches have also begun to disclose some breakthroughs in efficient 2D-based inter-frame point cloud compression. For example, Tu et al. [86] explored a U-net-based deep learning network for the real-time compression of point clouds stream. Using raw data from LiDAR sensors, the point cloud information is stored in a 2D matrix, being next converted to a video-like format. In this format, each row contains a laser ID, and each column represents laser beam emissions with the same vertical resolution, being the distance information defined by the values in each cell. Some frames are then designated as references and go through an interpolation process with the remaining using the U-net [87] architecture, a well-established image segmentation convolutional network. The final steps consist in calculating the residuals between the predicted and the actual frames, and applying an encoder network to further compress these values. In order to retain as much information as possible, the referenced frames are compressed using JPEG-LS. Similarly, Liu et al. [88] incorporated U-net for the inter-frame interpolation process in their approach. However, instead of using traditional codecs like JPEG-LS in the subsequent steps, the approach proposed by Wang et al. [75] is used to handle intra-frame compression. The method originally proposed by Sun et al. [67] was further improved with an inter-frame step [89]. Using a prediction network model based on convolutional Long Short-Term Memory (LSTM) cells, the algorithm can predict future frames, compressing the delta between them. LSTM are a type of RNN architecture designed to address the common vanishing gradient problem by allowing the network to selectively update and retain information over long sequences. Since LSTM effectively captures long-term dependencies, it is well suited for compressing sequential LiDAR data.

Some methods categorize their approach based on their frame structure, describing which frames they use to achieve data reduction. Commonly, there are three types of frames: intra-frames (I-frames), predictive frames (P-frames), and bidirectional frames (B-frames). The I-frames can hold a complete frame independent from the other frames. Often used as a reference, the I-frames are encoded independently. The P-frames rely on information from previous frames (typically I-frames or other P-frames) to predict and encode the changes that occurred since the last reference frame. Only the changes are stored, which helps reducing the data size. Finally, the B-frames can use information from both previous and future frames to predict and encode the changes. Focused on this last frame type, Zhao et al. [90] proposed a bi-directional frame prediction network for inter-frame prediction followed by a 32-bit high-precision floating-point lossy encoder to compress the I-frames and B-frames. Some mapping applications also propose algorithms to compress spatially and temporally point clouds. Wang et al. [91] developed a method relying on the plane fitting of RI’s segments retrieved from the RangeNet++ architecture [72]. To remove temporal redundancy, an interpolation-based network is used. Inspired by the PNG algorithm, the work proposed by Zhou et al. [92] uses an RI-based method with a deep model to predict the pixel values. Then, the predicted values and the original values to achieve lossless compression are compressed using a delta encoding approach. This method, rather than simply computing a difference between close-by pixels, calculates pixel values based on context pixels, achieved by LiDAR’s contextual laser shots on the raster-scanning order from both the current and past scans.

**Table 4:** Summary of 2D-based inter-frame compression methods.

Category	Method	Type	Main Features	Setup	Dataset	Performance	Source Code
Traditional	RI-LZW (1984) [80]	Lossy	Applies the LZW codec on a sequence of range images created from LiDAR	Intel Core i5-4210U	Velodyne HDL-64 sensor	PSNR: 63 [39]	Open source
	RI-MJ2 (2003) [60]	Lossy	Applies the MJ2 codec on a sequence of range images created from LiDAR	Intel Core i5-4210U	Velodyne HDL-64 sensor	PSNR: 63 [39]	Open source
	RI-H.264 (2014) [78]	Lossless	Applies the H.264 codec on a sequence of range images created from LiDAR	Intel Core i7-4770	Velodyne HDL-64 sensor	bpp: 2.41	Open source
	RI-LayerJPEG (2016) [81]	Lossy	Applies the JPEG codec to layered range images created from LiDAR	Not disclosed	Velodyne HDL-64 sensor	PSNR: 49–80	Not disclosed
	RT-ST (2020) [85]	Lossless	Uses iterative plane fitting to exploits both spatial and temporal redundancies	Intel Core i5-7500 and Nvidia mobile TX2	SemanticKITTI	CR: 40–90	Not disclosed
	PC-SLAM (2021) [82,83]	Lossy	Uses location and orientation information for LiDAR data compression	Intel Core i7-7820X	Velodyne HDL-64 sensor	bpp: 3.61–6.68	Not disclosed
	CLUSTER-ICP (2021) [84]	Lossless/Lossy	Uses CLUSTER [67], registration-based inter-prediction and lossless compression on residuals	i5-6300HQ 2.3 GHz w/ 4GB RAM	KITTI	CR: 9.47–41.49	Not disclosed
	FLiCR (2022) [79]	Lossy	Uses H.264 video codec on lossy RI for edge-assisted online perception	Nvidia Jetson AGX Xavier	KITTI	CR: 21.26–215.85	Not disclosed
Learning	RT-S-PCC-U-NET (2019) [86]	Lossless	Uses U-Net [87] to reduce temporal redundancies in a sequence of frames	Intel Core i7-7820X w/ Nvidia GeForce GTX 1080	Velodyne HDL-64 sensor	bpp: 2–4.5	Not disclosed
	Inter-Inserting (2022) [91]	Lossless	Uses plane fitting on RangeNet++ [72] RI’s segments and an interpolation-based network for temporal redundancy removal	Desktop w/ Nvidia TITAN RTX	KITTI	CR: 14.56–32.36	Not disclosed
	CLUSTER-LSTM (2022) [89]	Lossless/Lossy	Uses CLUSTER [67] for intra-prediction and convolutional LSTM cells for inter-frame compression	Intel 2.2GHz i7 w/ Nvidia GPU and 16GB RAM	KITTI	CR: 24.39–63.29	Not disclosed
	RIDDLE (2022) [92]	Lossy	Uses a deep model to predict the next pixel values based on current and past LiDAR scans and delta encoding to compress the data	Nvidia Tesla V100	Waymo Open and KITTI	bpp: 3.65–4.3	Not disclosed
	BPNet RAFC (2022) [90]	Lossy	Uses a frame prediction network to inter-frame prediction and floating-point lossy encoder for I- and B-frame residuals	Intel Core i7-7700K w/ Nvidia GTX 1080Ti and 16GB RAM	KITTI	bpp: 5.7–7.3	Not disclosed
	BIRD-PCC (2023) [88]	Lossless	Uses R-PCC [75] as intra-frame compression and U-Net [87] w/ a binary mask for inter-frame compression	Not disclosed	SemanticKITTI and KITTI-360	bpp: 1.7–4.2	Not disclosed

## 7. Three-Dimensional-Based Compression Algorithms

The 3D-based group includes techniques that break down the data provided by LiDAR sensors into smaller 3D data structures, improving the efficiency of compression techniques that take advantage of the partitioned 3D representations. The existing methods can be grouped into (1) tree-based, (2) sparse-tensor-based, and (3) point-based data structures.

### 7.1. Tree-Based

**Figure 8:**
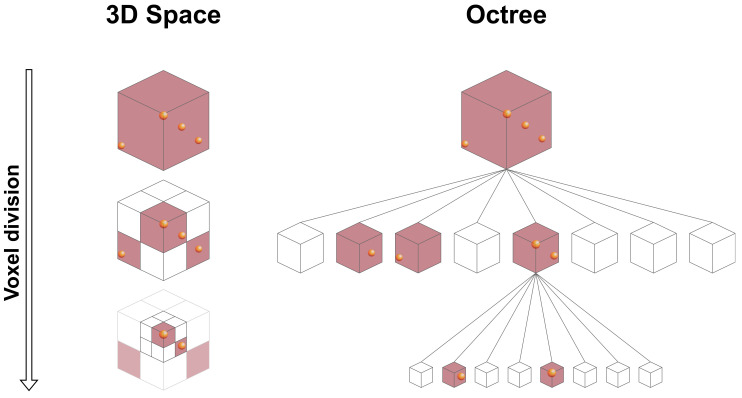
Octree and octants representation.

One of the most popular approaches for decomposing and compressing 3D point cloud data is employing space partitioning trees. An octree, which is the most well-known approach in this category [93], is a hierarchical data structure that organizes data in branches, each containing up to eight nodes, where each node can be itself a new branch or a leaf node. While branch nodes have children, leaf nodes represent an end-point in the octree. When applying an octree to 3D data, a node represents a cube or cuboid known as a bounding box, which can be subdivided into smaller cubes at each iteration to organize data. Firstly, the octree’s root defines a bounding box around the complete 3D dataset, representing the entire point cloud. Then, this bounding box is recursively subdivided into eight smaller boxes called octants, each corresponding to different regions of the 3D space, as shown in Figure 8. Finally, points from the point cloud are added to the octree by traversing the tree from the root to a leaf node, testing each node along the way to verify the point’s corresponding octant. The final data structure is then encoded using a compression technique that exploits the binary data resulting from the octree occupation. A summary of existing tree-based techniques applied to automotive LiDAR is presented in Table 5.

Octrees were firstly introduced to compress LiDAR data by Schnabel in 2006 [94]. The proposed method includes a lossless octree-based geometry compression technique for dense point clouds that decomposes the point cloud into smaller regions. In order to achieve this, the points are quantized to create octree cells with multiple sub-levels. Next, the method applies prediction techniques based on local surface approximations to find the non-empty child count of a cell, and based on this prediction, the child cell configuration. Finally, the predicted values are encoded using an arithmetic encoder. Kammerl et al. [95] proposed a real-time octree-based compression method that can be used for data streaming and storage. The approach consists of creating a differential octree from consecutive data frames of octree structures by applying an XOR operation. The resulting difference is then encoded using an integer arithmetic range encoder. Nardo et al. [39] and Anand et al. [96] also included octrees in their works to compress LiDAR data by exploring implementations provided by the PCL. However, the results are quite limited.

**Table 5:** Summary of 3D tree-based compression methods.

Category	Method	Type	Main Features	Setup	Dataset	Performance	Source Code
Traditional	PCL Octree compression (2011) [57]	Lossy	Offers 3 precision levels for PCL’s octree representation	Intel Core i5-4210U	Veloview Sample Dataset	CR: 1.85–2.81 [39]	Open source
	RT Octree XOR (2012) [95]	Lossy	Calculates the difference between consecutive frames by applying an XOR on octrees	Standard consumer PC	-	bpp: 0.38–0.88	Part of PCL
	RT Octree PCL Compression (2019) [96]	Lossless	Uses the PCL’s progressive 3D mesh coding to compress the octree	Nvidia Jetson TX2	KITTI and Ouster sensor	CR: 2.8–5.45	Open source
	G-PCC TMC13 (2020) [97]	Lossless/Lossy	Point cloud compression standard that uses octree voxelization and arithmetic coding	Not disclosed	SemanticKITTI	bpp: 1.4–4.9 [98] PSNR: 71–83 [98]	Open source
	Cylindrical RAHT (2021) [99]	Lossy	Uses cylindrical coordinates before the RAHT to predict the attributes of octree nodes	Not disclosed	KITTI and PandaSet	bpp: 20–23.7	Not disclosed
	VPO Inter-EM (2022) [100]	Lossless/Lossy	Improves Inter-EM’s global motion with a histogram-based point cloud classification based on vertically positioned objects	Not disclosed	Ford dataset	-	Not disclosed
	HM Inter-EM (2022) [101]	Lossless/Lossy	Uses Hamming distance between the octree’s nodes, instead of G-PCC geometric distance	Not disclosed	Ford dataset	bpp: 0.200–5.79	Not disclosed
Learning	OctSqueeze (2020) [102]	Lossy	Uses a tree-structured conditional entropy model to predict the probability of a symbol’s occurrence	Trained on 16 GPU	SemanticKITTI and NorthAmerica	bpp: 3.17–14.33	Open source
	MuSCLE (2020) [103]	Lossless	Uses a model to capture the spatial and temporal relationships between data points	Trained on 16 GPU	UrbanCity and SemanticKITTI	bpp: 4.68–18.47	Not disclosed
	VoxelContext-Net (2021) [104]	Lossless	Uses a combination of octree decomposition, entropy coding, and spatial context information	Nvidia 2080TI	SemanticKITTI	bpp: 0.207–5.93	Not disclosed
	OctAttention (2022) [105]	Lossless	Gathers sibling/ancestor node information to encode octree symbol sequences	Xeon E5-2637 w/ Nvidia TITAN Xp and 12G RAM	SemanticKITTI	bpp: 0.13–3.740	Open source
Learning	PCC-SC-SP (2022) [106]	Lossless/Lossy	Exploits quadratic surfaces and octree’s hierarchical dependency on siblings’ children, ancestors, and neighbors	2 Nvidia 3090	KITTI and nuScenes	bpp: 0.15–3.8	Not disclosed
	EHEM (2023) [107]	Lossless	Uses a similar attention encoding model as [105] but with a structure more friendly for parallel processing	2 AMD EPYC 7742 w/ 8 Nvidia A100	Ford and SemanticKITTI	bpp: 0.10–2.990	Not disclosed
	ECM-OPCC (2023) [108]	Lossless/Lossy	Uses segmentation and a dual transformer setup to find connections between nodes and their ancestors and siblings	Nvidia A100-PCIE 40GB and Nvidia GeForce RTX 3090	SemanticKITTI	bpp: 0.12–2.740	Not disclosed
	SCN (2023) [109]	Lossless	Leverage sparse 3D convolutions to extract features at various octree scales	Not disclosed	Ford and SemanticKITTI	bpp: 17.5–20.5	Not disclosed
	ML-GEM (2023) [110]	Lossless	Constructs the octree entropy model in layers, utilizing a variable to encapsulate the sibling/ancestor dependence	Nvidia GeForce RTX 3090 and 24GB RAM	Ford and SemanticKITTI	bpp: 0.2–3.8	Not disclosed

**Figure 9:**
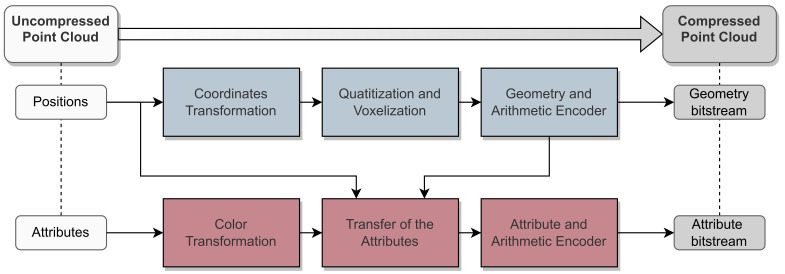
G-PCC method stages.

With the growing interest in compressing 3D point cloud data, the Moving Picture Experts Group (MPEG), a renowned alliance known for developing video standards, established the 3D Graphics Coding Group (3DG) group to study and propose new standards specifically tailored for 3D point clouds. Initially, these standards primarily addressed computer-animated content and were not suitable for real-time or real-world scenarios, typically involving well-structured and denser point clouds. Later, in 2017, a call for proposals (CfP) was launched, leading to the development of three new algorithms [111]: (1) S-PCC for static point cloud data; (2) L-PCC for dynamically acquired data; and (3) V-PCC for dynamic content. The two standards released in 2020 by the MPEG alliance were the video-based V-PCC, suitable for point clouds with a more uniform point distribution, and the G-PCC, a combination of the other two proposed standards (S-PCC and L-PCC) targeting sparser point clouds. G-PCC has been proven to be the most suitable for automotive LiDAR data compression [112]. The G-PCC standard defines a lossy scheme with lossless support, employing a geometry-based approach that can handle a wide range of cases, including both sparse and dense point clouds, as well as dynamic and static objects [97,113]. The points’ geometry and attributes are compressed separately and sequentially, as attribute compression directly depends on geometry. Initially, G-PCC transforms the points within the point cloud from floating-point to integer format. This transformation reduces the precision of the point cloud data but enables voxel representation using fewer bits and, consequently, achieves higher compression ratios. Subsequently, the geometry values are encoded using an octree-like voxelization followed by arithmetic coding. In the final step, G-PCC employs a configurable prediction method followed by arithmetic coding to compress the attributes. Figure 9 illustrates the overall compression flow of G-PCC.

Given the significance of G-PCC in point cloud compression, several studies have been conducted to evaluate its performance under various conditions and proposed new approaches and enhancements to the method. For instance, Garrote et al. [98] evaluated the G-PCC standard with the SemanticKITTI dataset [114], while Sridhara et al. [99] evaluated G-PCC’s Region Adaptive Hierarchical Transform (RAHT) step against their proposed cylindrical RAHT approach. The traditional RAHT method operates within Cartesian coordinates, initiating by segmenting the point cloud into regions. Each region undergoes independent transformation using a hierarchical approach, starting from the root (the entire point cloud) and cascading down to individual points. In contrast, the cylindrical RAHT approach employs cylindrical coordinates to represent points, capitalizing on the unique characteristics of LiDAR point clouds resulting from circular scanning trajectories. This adaptation allows for attribute encoding based on a volumetric partition in cylindrical coordinates, extending the capabilities of the RAHT step.

**Figure 10:**
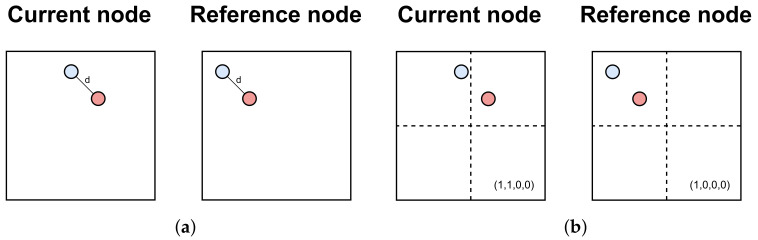
Inter-EM distortion prediction and the method proposed by An et al. [101]. (**a**) Distortion prediction based on the distance *d* between two adjacent points. (**b**) Distortion prediction based on points occupancy within the node.

Furthermore, the MPEG group enhanced the G-PCC standard with the inter-EM extension to address temporal relationships between points in consecutive frames, thereby improving efficiency with better inter-prediction for geometry and attributes. Typically, points within LiDAR point clouds exhibit two distinct types of movement: global motion, influenced by the vehicle where the sensor is mounted, and local motion, generated by surrounding dynamic objects such as other cars. Inter-EM leverages these motions to achieve higher compression ratios. To further enhance the global motion estimation of Inter-EM, Kim et al. [100] introduced a histogram-based point cloud classification that considers both vertically and horizontally positioned objects, in contrast to Inter-EM’s single horizontal classification. The traditional Inter-EM approach evaluates the point distance *d* within octree nodes to detect changes (as depicted in Figure 10a). However, if the distance *d* between two points remains the same, the distortion is not detected. Targeting a different part of the inter-EM inter-frame step, An et al. [101] introduced a new approach to improve distortion prediction. This method subdivides the node into smaller sub-nodes to evaluate the point’s occupancy (as shown in Figure 10b), addressing additional frame changes, even when point distances remain constant between frames.

**Figure 11:**
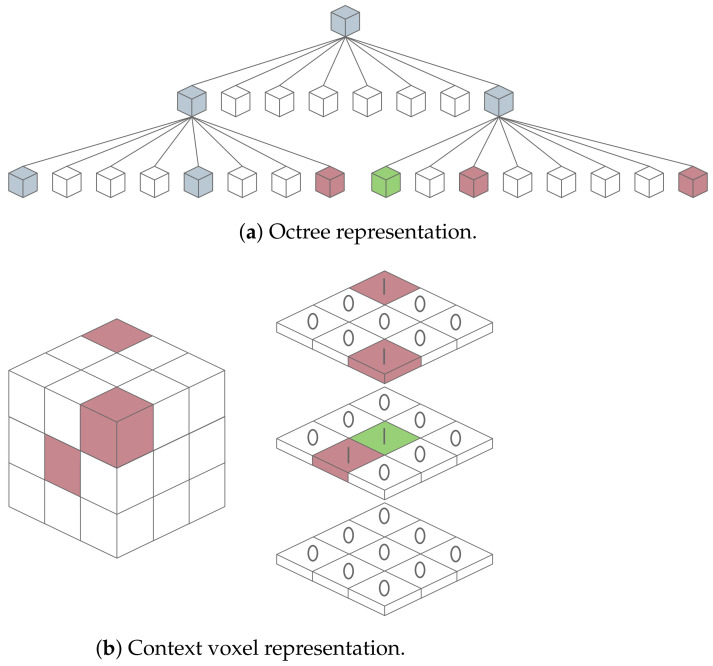
Hybrid structure based on octree and context voxel. Colorful cuboids represent nodes with points: Green—point under evaluation; Red—neighbors; and Blue—other nodes.

Three-dimensional learning-based techniques also start to emerge for automotive LiDAR data compression. Huang et al. [102] introduced one of the first deep learning-based lossy methods aimed at reducing the memory footprint caused by LiDAR sensors. The primary focus of this method is to leverage redundancies between points to minimize the bit rate. Initially, the raw point cloud is pre-encoded using octrees. Subsequently, using a conditional entropy model, the algorithm learns the tree structure and predicts the symbols of each intermediate node based on its 8-bit occupancy values (children cell occupancy code). The predicted probabilities are then fed into an entropy encoder, which converts the serialized symbols into a final bitstream. Biswas et al. [103] proposed a deep entropy model that also operates on octree structures. However, it differs from the previous work as it also addresses temporal redundancies between successive frames. Addressing the redundancy created by hierarchical dependencies often associated with octree-based techniques, Que et al. proposed an alternative method in their study [104], as illustrated in Figure 11. This approach involves creating a context voxel representation of each point’s neighborhood. By conducting a neighborhood search in the point cloud, it covers the possibility of having near points that are represented outside of their parent node.

Fu et al. [105] further improved the previous work by proposing a model with a greater context size, including in the voxel the sibling nodes and their ancestor’s information. To mitigate the overhead created by increasing the voxel size, Song et al. [107] adopted a similar hierarchical attention model but with a grouped context structure designed to enable parallel decoding. Also aimed at facilitating parallel processing, Jin et al. [108] proposed a segment-constrained multi-group coding strategy. Additionally, the method uses a dual transformer architecture based on the dependency of the current node on its ancestors and siblings, and a random-masking pre-train method to enhance the model. Chen et al. [106] addressed the inter-voxel redundancy present in context voxel-based methods by enriching the entropy model with information from decoded siblings’ children, ancestors, neighbors, siblings, and siblings’ children. The work by Luo et al. [76] also adopts octrees for point cloud representation. However, the spherical coordinates system is used for the octree creation instead of the Cartesian coordinates. This leverages the prevalence of vast circular-shaped point chains within point clouds, a crucial feature of spinning LiDAR sensors. To compress redundant information in the point cloud, points from the same chain are grouped into the same voxel, increasing the efficiency of the method. This logic suggests that the spherical coordinate system could potentially outperform both Cartesian and cylindrical coordinates by considering the azimuthal angle invariance of points. To demonstrate the effectiveness of their approach, Luo et al. applied their pre-processing step to the works of Song et al. [107] and Fu et al. [105]. On the other hand, instead of utilizing a regular 3D convolution architecture like OctSqueeze [102] and VoxelContext-Net [104], Lodhi et al. [109], and Fan et al. [110] proposed sparse 3D convolution architectures in octree representations. While the former extracts features in multiple octree scales to capture feature propagation from ancestor to sibling nodes, the latter constructs the octree entropy model hierarchically in layers and incorporates a variable as side information to account for sibling and ancestor dependencies. This enables high parallelization, as each layer can be decoded independently.

### 7.2. Sparse-Tensor-Based and Point-Based

**Figure 12:**
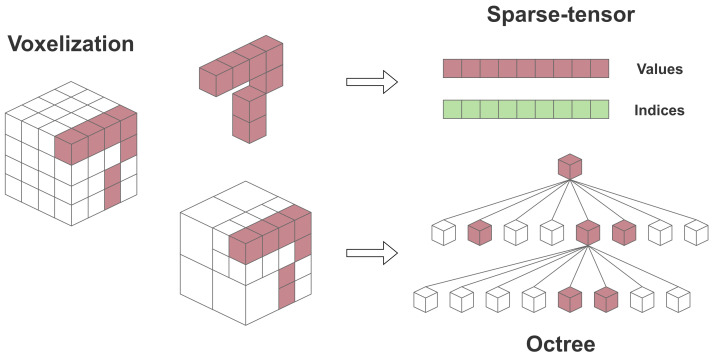
Octree and sparse-tensor visual representation.

Recognizing the complex nature and computational demands of tree-based methods, some works have shifted their focus towards alternative approaches for representing LiDAR data. Considering the sparsity of a point cloud and that its representation predominantly contains empty (zero) values, sparse-tensors can be used as an efficient data structure to store them. In contrast to octrees, which are designed to represent 3D data hierarchically, tensors can represent multi-dimensional data in a compressed format where only the non-zero entities are stored with their corresponding index values, as depicted in Figure 12.

One of the first works suggesting a multi-scale sparse tensor representation for sparse convolutions was presented by Wang et al. [115]. This method divides the point cloud into voxels, using a binary occupancy status to describe which voxels are occupied or empty. The voxels containing points, called Positively-Occupied Voxels (POVs), are then progressively downscaled, being the occupancy probability encoded into the binary stream at each iteration. For the decoding process, a SparseCNN-based Occupancy Probability Approximation (SOPA) model is used to retrieve the Most-Probable-POVs (MP-POVs) from each POV’s occupancy probability. In each scale, MP-POVs are then classified into POVs or empty voxels, recursively upscaling the point cloud. The complexity is significantly reduced since the SOPA is only used in POVs, and those are represented as sparse-tensors. Xue et al. [116] proposed a different method also targeting sparse-tensors. Instead of staking 3D sparse convolutions for neighborhood spatial correlations as in the SOPA model, it is used a Neighborhood Point Attention (NPA) to construct an adaptive local neighborhood and leverage it to dynamically aggregate neighborhood information. Therefore, the proposed Multistage Occupancy Probability Approximation (MOPA) model acts similarly to the SOPA, but it combines NPA and sparse convolutions layers to achieve better performance.

Another approach to handle point clouds is to use features directly retrieved from points, avoiding discretization effects caused by any representation. Wiesmann et al. [117] use kernel point convolutions (KPConvs) to extract local feature descriptors. KPConvs, firstly by introduced Thomas et al. [118], define a continuous convolution for unordered point clouds, learning a weight for each position in a set of kernel points. Designed for segmentation and classification, this method handled downsampled lost points with skip connections, a type of architecture where the output of one layer is fed to layers further down the network. However, this approach is unsuitable for point cloud compression as skip connections would require storing intermediate results for the decoder component, making the whole process inefficient. Therefore, Wiesmann et al. proposed a decoder that uses continuous 3D deconvolutions to recover the points based on learned features. Mari et al. presented a framework [119] that exploits semantic information within point clouds to optimize data transmission. Similarly to other semantic-based methods, they first segment data into separate streams. Then, each cluster is independently coded, allowing the tuning of its compression parameters according to the significance of its coded class and the desired compression level. Furthermore, for applications that require semantic segmentation after the compression/decompression process, segment information can also be coded in the compressed stream, alleviating not only the receiver system but also reducing the distortion introduced by semantic segmenting a point cloud reconstructed from a lossy stream. The framework uses the RandLA-Net [120], a lightweight point-based architecture designed for sparse point clouds, in the first step. Then, the segments are encoded using one of the already established codecs for point cloud compression, such as MPEG’s GPCC TMC13 implementation and Google’s open source library Draco [77]. Table 6 summarizes the current sparse-tensor-based and point-based methods.

## 8. Discussion

With the vast number of methods present in the literature, choosing the best approach to compress LiDAR data in automotive environments requires a crucial understanding of the application requirements and the trade-offs that each approach offers.

### 8.1. Performance Metrics

Table 7 summarizes a qualitative comparison between each group of methods available in the proposed taxonomy considering the following metrics: real-time processing, computational requirements, compression level, distortion, adaptability, scalability, and the final application goal. This comparison is made solely based on the information available in the literature, whose experimental setups were not validated by this work.

**Real time:** In the context of autonomous driving applications, it is crucial for the perception system to swiftly understand the surrounding environment in real time. Therefore, the steps involved in LiDAR’s processing stack must be executed within a specific time frame to ensure that driving decisions can be made safely. Considering that high-resolution LiDAR sensors are capable of generating millions of points per second, the compression methods that have the goal of reducing data for applications such as object detection, segmentation or tracking, must guarantee that the approach can help improve the overall processing time. Since most automotive LiDAR sensors operate at 10 Hz, the considered deadline for real-time processing is 100 ms, which is hard to achieve with complex algorithms. With the emergence of new sensor technologies that can provide point cloud frames at higher rates, e.g., 20 Hz, the real-time metric will be harder to achieve. From the taxonomy presented in Figure 3, most coding-based methods can achieve this metric due to their low complexity. With the usage of standard image codecs in range-image representations, some traditional 2D intra-frame-based approaches can also achieve real-time processing when using lower compression levels and lossy compression. However, for the remaining groups, real-time processing can only be achieved when only specific segments of the data are compressed.
**Computational requirements:** Aiming to achieve optimized SWaP-C requirements, perception systems are often composed of processing units with limited resources. Thus, it is desirable that a compression method suits resource-constrained platforms. Regarding the coding-based compression methods, they have the lower computational requirements from all groups. These approaches can be deployed either within the sensor, thereby minimizing on-chip memory usage, or in close proximity to the sensor during an early processing step. The format-based methods can be considered simple after the point cloud is converted to the required format. Nonetheless, this conversion can require considerable resources if placed close to the sensor. Due to the well-established codecs and application-specific accelerators deployed in hardware, image-based compression presents medium to high computational requirements. This is because the group is the one that offers the widest variety of methods, ranging from simple approaches focused on achieving low latency to others more tailored to achieve better compression ratios. While video-based compression is a well-established research domain, it demands an inter-frame step in addition to intra-frame compression, which can lead to increased processing power requirements. On the high end of the spectrum, 3D-based methods are the ones that require more powerful computational resources. Octree, sparse, and point representations usually require a big memory footprint when compared to 2D representations, especially when learning-based methods are deployed to model point clouds. In addition to the training process, which usually requires high-end computers to speed up the process, learning methods usually demand powerful graphics processing units (GPUs) to reach their full potential.
**Compression level and data Distortion:** The compression level refers to the algorithm’s ability to reduce the size of LiDAR data, while distortion indicates how close to the original are the resulting data are after being decompressed, which can be caused by lost data or estimations during the compression process. Metrics like CR and bpp can help characterize the methods’ behavior under a well-defined setup. However, they can vary according to the environment where data were collected. This is particularly evident for learning-based methods as these require training data, significantly affecting their overall performance. The methods that provide the highest compression rates are 3D-based and 2D inter-frame-based, as they excel in understanding the relationship between points and existing redundancies in the point cloud information. Their distortion level can vary from none (lossless configurations) to very high (very lossy approaches). Nonetheless, tree-based methods, mainly due to their hierarchical representation, currently achieve the best trade-off between compression level and distortion. On the other hand, the 2D-based compression methods offer medium to very high compression levels when inter-frame compression is also used. Despite some methods providing lossless compression, which does not cause distortion, the most prominent use lossy range-image representations before applying lossless methods. Therefore, range images can be compressed/decompressed without losing information, but point cloud data cannot be accurately restored to its original version. Finally, coding-based and format-based approaches offer very low compression levels due to their simplicity, as their primary goal is not to create distortion.
**Adaptability and scalability:** With the constant technological evolution around LiDAR sensors, current algorithms will face different challenges to adapt to newer point cloud representations or data structures. Coding-based algorithms rank highest in adaptability since these process data as bytes, regardless of the sensor type or data representation. Following closely are 3D point-based algorithms, which handle sensor data as points in the 3D space, remaining effective as long as the sensor provides data in a point-based format. The remaining 3D-based and 2D-based categories typically require a pre-processing step to achieve the necessary data representation for compression. While still adaptable, these methods require some tuning in their pre-processing steps to align with sensor outputs and method requirements. Lastly, format-based algorithms rank lowest in adaptability, as these require more steps to achieve the data structure required by the compression algorithm. Among these, the LAS-based algorithm is considered the least adaptable, since the LAS format was specifically designed for airborne sensors and applications.

Modern sensors tend to provide point clouds with higher resolutions at higher frame rates. Regarding the scalability features of the proposed methods, and not considering the processing latency related to the increasing number of points, 3D-based methods would still perform well with denser point clouds. Methodologies that extract shapes to perform compression can swiftly discard redundant points as the point cloud density increases. For example, methods utilizing octree structures can efficiently discard redundant points once the leaves are already occupied. Next in line are the 2D-based methods, as they operate on similar principles but in a 2D context. Some of these methods can also identify redundant points across multiple frames, which is particularly advantageous with higher frame-rate sensors. The performance of the remaining methods is proportional to the size of the data they compress. Consequently, they do not benefit from an increase in data volume. It is worth noting that dictionary-based methods could potentially exhibit a superior performance compared with the remaining others because point clouds with more points often result in patterns already stored in the dictionary. As a result, the dictionary size does not increase linearly with the number of points, leading to higher compression rates.

**Compression goal:** According to the real-time capabilities and the compression ratios a method can achieve, it would better suit applications that require data streaming, storage, or both. Methods targeting stream compression must be capable of providing real-time performance, even if the compression ratio or regions in the point cloud are sacrificed. Conversely, storage methods aim to achieve higher compression ratios sometimes requiring more complex computational requirements and considerable processing time. The groups capable of providing data compression for stream applications are the coding-based and 2D Intra-frame. Tree-based methods can also be included in this category, as some of the methods within this group are attempting to reduce latency to achieve real-time performance. The remaining groups either have high processing latencies, or are primarily focused on achieving high compression ratios, which may indicate that they are more suitable for data storage applications. With the rapid increase in learning-based methods, which heavily rely on offline data for training, these approaches are also mainly focused on storage compression.

### 8.2. Future of Automotive LiDAR Data Compression

LiDAR sensors are rapidly solidifying their presence in systems with automation features classified as level 2 and level 3 [121]. However, achieving fully autonomous driving features remains a distant goal, as these depend on real-time multi-sensor setups that rely on huge volumes of data to work properly. While some perceive LiDAR sensors as expensive components that are complex to manage, others see them as crucial for advancing the autonomous driving technology, putting several research efforts into handling the data output from several LiDAR sensors present in the perception system. Data compression becomes a crucial step in handling the LiDAR sensor’s output; however, due to the ongoing evolution of this technology, both in terms of data representation and data size, not all presented methods will be able to adapt. From the methods present in the proposed taxonomy, 3D-based approaches, such as octree-based algorithms and standard methods, such as G-PCC, are more likely to be able to follow the evolution of LiDAR technology. While octrees scale better, standard representations often face worldwide adoption due to the interoperability and adaptability features they offer.

Exploring potential breakthroughs in LiDAR compression could require a multifaceted approach encompassing advancements in hardware and software designs. Dedicated hardware tailored for assisting or fully supporting the compression tasks, such as Application-specific Integrated Circuit (ASIC) and field-programmable gate array (FPGA)-based solutions, can help in reducing compression latencies and enhancing overall system performance, which could be a promising solution for hosting and improving 3D learning-based algorithms. Furthermore, AI-based approaches empowered with tensor processing units (TPUs) and neural processing units (NPUs) are already driving innovation across various applications. Leveraging such hardware capabilities alongside more suitable data representations, such as sparse tensors, point-based, or tree-based structures, can represent an alternative solution for handling the ever-growing LiDAR data output.

Given its applicability, LiDAR compression cannot exist standalone as it must interact seamlessly with other critical automotive functionalities. For instance, efficient compression can help ensuring that object detection algorithms receive precise and timely data, underscoring the importance of balancing compression ratios with detection performance. Sensor fusion, which involves integrating LiDAR data with radar, cameras, and GPS, requires holistic system optimization for future advancements, where LiDAR compression will play an important role in the integration of all these data sources. Moreover, real-time processing and energy efficiency are paramount for autonomous vehicles, demanding swift compression algorithms that minimize latency and power consumption, thereby extending the vehicle’s absolute range, increasing system efficiency and improving its overall safety.

## 9. Conclusions

Integrating LiDAR sensors into perception systems presents a crucial advancement in autonomous driving technology. While LiDAR sensors offer unparalleled capabilities in generating high-quality point clouds for enhanced environmental perception, the challenge still lies in managing the substantial data output. The desire to improve sensor characteristics, especially point cloud resolution, results in increased volumes of data, posing significant challenges in handling, transmitting, and storing this information within complex yet resource-limited platforms such as those used in modern vehicles. Addressing these challenges calls for innovative compression solutions specifically designed for LiDAR data. Such solutions must consider a delicate balance between maintaining data integrity and meeting the tight requirements of autonomous driving applications.

This survey summarizes the existing methods, proposing a taxonomy to help understand the current trends in the automotive LiDAR compression research field. The algorithms are divided into four main categories: (1) coding-based; (2) format-based; (3) 2D-based; and (4) 3D-based methods—highlighting their main characteristics and approaches used to reduce data size according to the final application. Furthermore, it presents a qualitative comparison between all groups, highlighting their main features and considering the most important metrics, such as real-time, computational requirements, compression level, distortion, and compression goal. It finishes with some future insights on how the rapid evolution of LiDAR technology may affect existing methods and which groups are more likely to leave their mark as a reference for LiDAR data compression.

## Figures and Tables

**Table 1:** Summary of coding-based compression methods applied to LiDAR data.

Category	Method	Type	Main Features	Setup	Dataset	Performance	Source Code
Entropy Encoding	Golomb–Rice coding (1971) [42]	Lossless	Applies the Golomb–Rice algorithm to a stream of LiDAR data	Matlab simulation	LiDAR laboratory setup	CR: 1.01 [43]	Open source
Dictionary-based	LZMA (1998) [44]	Lossless	Applies LZMA algorithm to a stream of LiDAR data	Suggested in [45] to be used with LiDAR data, but not yet tested			Open source
Delta Encoding	SSDE (2016) [46]	Lossless	Adds symmetric and segment properties to delta encoding method	Matlab simulation	LiDAR laboratory setup	CR: 1.19–1.39 [43]	Not disclosed
	EDC (2019) [43]	Lossless	Adds overshooting detection to delta encoding method	Matlab simulation	LiDAR laboratory setup	CR: 1.61–1.89	Not disclosed
Bitmasking	V2I/V2V EC (2019) [47]	Lossless/Lossy	Exploits unnecessary precision by zeroing least significant bits	Intel Xeon E5-2620, Odroid XU and Raspberry Pi 3	Velodyne VLP-16 sensor	CR: 1.37–2.10	Not disclosed

**Table 2:** Summary of format-based compression methods.

Category	Method	Type	Main Features	Setup	Dataset	Performance	Source Code
LAS	LASzip (2013) [55]	Lossless	Compress the LAS format standard	Not disclosed	Velodyne HDL-32E and Ibeo sensors	bpp: 21.6–36 [58]	Open source
PCD	PCD compression (LZF) (2011) [57]	Lossless	Compress the PCD format standard binary with LZF	Not disclosed	Velodyne HDL-32E and Ibeo sensors	bpp: 81.6–96.8 [58]	Open source

**Table 6:** Summary of existing sparse-tensor-based and point-based methods.

Sub-Group	Method	Type	Main Features	Setup	Dataset	Performance	Source Code
Sparse Tensor-based	SparsePCGC (2022) [115]	Lossless/Lossy	Uses multiscale sparse tensors as the representation for their convolutions	Intel Xeon Silver 4210 w/ Nvidia GeForce RTX 2080	Ford and SemanticKITTI	bpp: 6.13–21.16	Open source
	GC-NPA (2022) [116]	Lossless/ Lossy	Uses NPA to aggregate information about the geometric correlations between points	Intel Xeon 6226R w/ Nvidia GeForce RTX 3090	Semantic KITTI and Ford	bpp: 4.78–12.80	Not disclosed
Point-based	DC-DPCM (2021) [117]	Lossy	Uses a convolutional autoencoder based on the KPConv to retrieve local feature descriptors	3.5 GHz Intel w/ Nvidia GeForce RTX 2080 SUPER	SemanticKITTI and nuScenes	bpp: 0.16–0.44	Open source
	CACTUS (2023) [119]	Lossy	Uses TMC13/Draco compression codecs on RandLA-Net [120] semantic segments	Not disclosed	SemanticKITTI	ΔPSNR: 0.98–3.52	Open source

**Table 7:** Qualitative comparison between taxonomy groups.

Group	Sub-Group	Real-Time	Comp. Reqs.	Comp. Level	Distortion	Adaptability	Scalability	Goal
Coding-based	Entropy encoding	✓	Very low	Low	None	Very high	Medium	Stream
	Dictionary-based	✓	Very low	Low	None	Very high	High	Stream
	Delta encoding	✓	Very low	Low	None	Very high	Medium	Stream
	Bitmasking	✓	Very low	Low	Low	Very high	Medium	Stream
Format-based	LAS	-	Low to medium	Low	None	Medium	Medium	Storage
	PCD	-	Low to medium	Very Low	None	Medium	Medium	Storage
2D-based	Intra-frame	✓	Medium to high	Medium to high	None to medium	High	High	Stream/Storage
	Inter-frame	✗	High to very high	High to very high	None to medium	Medium	High	Storage
3D-based	Tree	✗	High to very high	High to very high	None to medium	High	Very high	Stream/Storage
	Sparse-Tensor	✗	High to very high	High	None to medium	High	Very High	Storage
	Point	-	High to very high	Very high	Medium to high	High	Very high	Storage
